# Unlocking the Potential: Epstein-Barr Virus (EBV) in Gastric Cancer and Future Treatment Prospects, a Literature Review

**DOI:** 10.3390/pathogens13090728

**Published:** 2024-08-28

**Authors:** Salvatore Corallo, Angioletta Lasagna, Beatrice Filippi, Domiziana Alaimo, Anna Tortorella, Francesco Serra, Alessandro Vanoli, Paolo Pedrazzoli

**Affiliations:** 1 Department of Internal Medicine and Medical Therapy, University of Pavia, 27100 Pavia, Italy; beatrice.filippi02@universitadipavia.it (B.F.); domiziana.alaimo01@universitadipavia.it (D.A.); anna.tortorella01@universitadipavia.it (A.T.); f.serra@smatteo.pv.it (F.S.); p.pedrazzoli@smatteo.pv.it (P.P.); 2 Department of Oncology, Fondazione IRCCS Policlinico San Matteo, 27100 Pavia, Italy; a.lasagna@smatteo.pv.it; 3 Department of Molecular Medicine, University of Pavia, 27100 Pavia, Italy; a.vanoli@smatteo.pv.it; 4 Anatomic Pathology Unit, Fondazione IRCCS Policlinico San Matteo, 27100 Pavia, Italy

**Keywords:** gastric cancer, epstein-barr virus, biomarker, molecular classification, treatment options

## Abstract

Gastric cancer (GC) is a complex disease with various etiologies. While *Helicobacter pylori* infection is still one of the leading risk factors for GC, increasing evidence suggests a link between GC and other infective agents such as Epstein Bar Virus (EBV). EBV-associated gastric cancer (EBVaGC) is now recognized as a distinct subgroup of GC, and the complex interactions between the virus and gastric mucosa may influence its development. A recent integrative analysis of the genome and proteome of GC tissues by The Cancer Genome Atlas project has identified EBVaGC as a specific subtype characterized by *PIK3CA* and *ARID1A* mutations, extensive DNA hyper-methylation, and activation of immune signaling pathways. These molecular characteristics are markers of the unique molecular profile of this subset of GC and are potential targets for therapy. This review aims to provide an overview of the current knowledge on EBVaGC. It will focus on the epidemiology, clinic-pathological features, and genetic characteristics of EBVaGC. Additionally, it will discuss recent data indicating the potential use of EBV infection as a predictive biomarker of response to chemotherapy and immune checkpoint inhibitors. The review also delves into potential therapeutic approaches for EBVaGC, including targeted therapies and adoptive immunotherapy, highlighting the promising potential of EBV as a therapeutic target.

## 1. Introduction

Gastric cancer (GC) is the fifth cause of cancer death worldwide [1]. Increasing evidence indicates that *Helicobacter pylori* (*H. pylori*) infection is one of the leading causes of GC, along with smoking, alcohol consumption, and obesity [2]. However, *H. pylori* is not the only biological agent related to GC development. Evidence suggests that Epstein Bar Virus (EBV) infection has a causal link with the development of gastric malignancies, even though clear epidemiological evidence of a direct causal role is currently lacking [3].

EBV is one of the first viral agents to be associated with human malignancies [4]. Several studies have revealed that EBV could be related to various malignancies, including nasopharyngeal carcinoma (NPC), Hodgkin’s lymphoma, extranodal natural killer/T-cell lymphoma, and lymphoproliferative disorders of immunocompromised hosts [5].

The link between EBV infection and GC was first described in 1990 by Burke et al. They reported finding EBV DNA by polymerase chain reaction (PCR) in a case of undifferentiated gastric carcinoma with intense lymphoid infiltration [6]. Since then, several studies have confirmed the connection between EBV and a specific type of GC known as lymphoepithelioma-like carcinoma (LELC), which shares microscopic similarities with nasopharyngeal lymphoepithelioma [7,8,9,10]. Two years after this initial discovery, Shibata and Weiss explored the possibility of EBV presence in typical GCs, detecting EBV sequences in 16% of cases in a small North American study [11]. More recently, The Cancer Genome Atlas (TCGA) network identified EBVaGC as a distinct subgroup, comprising less than 10% of all cases and characterized by DNA hypermethylation, PIK3CA mutations, and activation of immune signaling pathways [12].

In this review, we offer a comprehensive analysis of the molecular, clinicopathological, and therapeutic aspects of EBVaGC based on the latest evidence. We also explore the potential of EBV infection as a predictive biomarker for response to specific therapies. Additionally, we examine ongoing studies and potential future treatment strategies specifically designed to leverage the growing body of evidence on the distinct genetic characteristics of this subset of GC.

## 2. Epidemiology

The distinct histologic features of LELC make it easily distinguishable from ordinary gastric adenocarcinoma. Although rare, the epidemiology and clinical characteristics of this subtype of GC have been well described. Unlike Burkitt’s lymphoma and nasopharyngeal lymphoepithelioma, which are endemic in equatorial Africa and Southeast Asia, respectively [13,14], EBVaGC is a non-endemic disease found worldwide [15]. Approximately 1–4% of all gastric cases are LELC, and large series suggest that EBV DNA can be detected in 80–90% of LELCs by PCR and in-situ hybridization (ISH) [8,16,17]. However, the prevalence of EBV in LELC appears to be higher in eastern Asia (82.5%), compared to western countries (29.5% in Italy, Portugal, United States) [17].

On the contrary, the global burden of EBVaGC among conventional adenocarcinoma is challenging to estimate due to the lack of routine EBV in GC cases worldwide, especially in the metastatic setting. In The Cancer Genome Atlas (TCGA) project dataset, EBVaGC accounted for 8.8% and 15% of the localized and metastatic GC cases sequenced, respectively [12]. However, due to the low number of metastatic GC patients included in the analysis (20 cases), the TGCA does not provide a reliable prevalence rate of EBVaGC. Recent meta-analyses estimated a global prevalence of EBVaGC of about 7.5–8.8% [15,18,19,20] and a prevalence of 7.5% among conventional adenocarcinomas. However, only a few studies included in these analyses could distinguish between LELC and non-LELC cases. These studies showed that the prevalence of EBV involvement in LELC is significantly higher than that for non-LELC (86.4% versus 6.1%) [18]. Moreover, among advanced-stage GC patients, the prevalence of EBV positivity is much lower (about 3–4%) than reported in limited-stage GC series [21,22]. Based on these data, about 81,000 conventional gastric adenocarcinomas are potentially attributable to EBV worldwide [18].

## 3. Pathogenesis

EBV is a double-stranded DNA Human Herpes Virus (HHV-4) that belongs to the subfamily of Gammaherpesviridae. Its circular double-stranded genome is approximately 172 kilobases and includes genes coding for almost 85 proteins and around 50 non-coding RNAs [23].

EBV chronically infects 90% of the adult population worldwide and its transmission occurs through saliva [24]. In developing countries, the first infection happens during childhood because of overcrowding and pre-chewing food [25]. In developed countries, the infection hits adolescents due to the exchange of saliva during intimate oral contact [26]. The primary infection can be associated with fever, pharyngitis, and lymphadenopathy, an illness called ‘Infectious Mononucleosis’ [24].

EBV has a biphasic lifecycle delineated into two phases: latency and lysis. The initial lytic phase is associated with primary infection and leads to the constitution of virions. During the infection, EBV releases its circular episome into the host cell. The episome can duplicate simultaneously with the host cell genome using host enzymes. The plasmid segregation in daughter cells is granted by a viral protein (Epstein–Barr nuclear antigen 1 [EBNA-1]) that makes the plasmid tethered to the host genome [27]. The latent phase, instead, is related to the presence of the viral genome in host cells without virus production. EBV genes expressed during the latent cycle are limited, and this characteristic of the EBV latent phase permits the avoidance of the host immune system and increases survival and pathogenesis [28]. Intermittent lysis can interrupt latency to amplify the infectious viral progeny [29].

Latency and its reversibility are important peculiarities of EBV infection. They allow the persistence of the viral genome in host cells and the activation in specific host conditions of immunodeficiency [30]. A state of host immunodeficiency may reactivate EBV-infected B cells, enabling them to infiltrate other mucosal sites where B cells are present [31]. EBV can re-establish its latency when entering the newly infected cells.

The most common question is about how EBV can access the gastric mucosa. EBV first infects oral epithelial cells in the tonsil and B cells in the lymphatic tissues of Waldeyer’s Ring; then, EBV establishes latent infection in lymphocytes, inducing proliferation of the infected cells [26]. When EBV-infected B cells differentiate into plasma cells and enter the lytic phase, EBV can return to the oropharynx for transmission through saliva [26,32]. It is possible that EBV present in ingested saliva can withstand the acidic environment of the stomach, allowing it to infect the inflamed mucosa [33]. Chronic inflammation is probably the key because it enables the arrival of lymphocytes. The epithelial cells secrete vesicular products that induce virus production in EBV-infected B cells, increasing the risk of infection of gastric tissue [34]. Some evidence demonstrates that the co-infection of EBV and *H. pylori* increases the risk of GC, and a recent study indicates that EBV enters epithelial cells through the ephrin A2 receptor, which is exposed by epithelial cells during gastric inflammation in *H. pylori* gastritis [35,36]. Indeed, both EBV and *H. Pylori* promote epithelial-mesenchymal transition, defined as a severe morphological and functional change of cells linked with dedifferentiation and invasion. It has been demonstrated that EBV causes long-lasting inflammation that can damage the gastric epithelium and promote precancerous lesions, such as Atrophic Gastritis, although less frequently than *H. pylori* [37].

The persistence of the infection and EBV interaction with cell DNA during replications can promote cancer development. However, the pathogenic mechanism of EBVaGC is still not completely understood. Analysis of the terminal repeat of the EBV genome in EBVaGC cases has shown that all tumor cells carry the same clonotype of the virus genome [38], suggesting that each EBVaGC is of monoclonal origin and supporting the theory of a cell tumor development from a single EBV-infected cell. The rare finding of EBV infection in precursor lesions (i.e., chronic gastritis, atrophic gastritis, and dysplasia) supports the hypothesis that EBV could play an early direct role in gastric carcinogenesis [39]. Conversely, it has been proposed that EBV infection is a late event in gastric carcinogenesis, occurring after the clonal expansion of a progenitor cell harboring several genetic/epigenetic alterations, has also been proposed.

In EBVaGCs, the viral genome is present in almost all carcinoma cells but is absent in surrounding normal gastric mucosa [11]. However, there have been reports describing cases where distinct EBV-positive and EBV-negative tumor areas coexist and cases of EBVaGC with intratumoral heterogeneous *EBER* expression [40,41,42,43,44]. One possible explanation for this is the disappearance of EBV infection in the intermediate and late stages of GC development in tumoral subclones rather than the coexistence of two distinct tumors with independent carcinogenesis. A recent study found that some cases of EBVaGC showed both EBER-positive and -negative components characterized by heterogeneous tumor cells with different viral loads and variable expression of viral transcripts but sharing common genetic/epigenetic alterations [42]. Although extremely rare, these cases may provide evidence supporting the hypothesis that EBV is eliminated from tumor cells during progression in these anecdotal EBV-positive and -negative collision tumors. The ‘hit-and-run’ theory is a fascinating mechanism in which certain viruses (such as herpesviruses, but also adenoviruses and papillomaviruses) can cause a transient infection, promoting the initiation of carcinogenesis (‘hit’) and leave behind epigenetic changes even after the virus has been eliminated (‘run’) [45]. Studies on the comprehension and demonstration of this theory are still in their infancy due to technical difficulties in studying the virome. Still, they could have diagnostic and therapeutic repercussions [46]. Recently, Siciliano et al. documented the presence of EBV infection in 17.5% of a cohort of 40 EBV-negative GCs by applying highly sensitive methods for EBV genome detection. In particular, they used droplet digital PCR (ddPCR) for EBV segments on microdissected tumor cells and RNAscope for EBNA1 mRNA as a confirmatory method [47]. This study does not provide direct proof of the hit-and-run theory but supports the concept that EBV can also be involved in gastric carcinogenesis in some cases of EBV-negative GC.

## 4. Genetic Features

Somatic gene alteration analyses revealed that EBVaGC frequently presents mutations in phosphatidylinositol-4,5-bisphosphate 3-kinase catalytic subunit alpha (*PIK3CA*) (80%), AT-Rich Interaction Domain 1A (*ARID1A*) (55%), BCL6 corepressor (*BCOR*) (23%), copy-number amplifications of Janus kinase 2 (*JAK2*) and *CD274*/*PDCD1LG2* (15%), and lack of *TP53* mutations [12,48].

*PIK3CA* regulates the PIK3/Akt pathway and is frequently mutated in cancers, including GC [48,49]. The PIK3/Akt pathway controls numerous cell activities, including cell proliferation, survival, and motility [50]. Most known *PIK3CA* mutations are located in exons 9 and 20, although multiple concomitant *PIK3CA* genotypes have been described [49,51]. The enrichment of non-silent *PIK3CA* mutations in EBVaGC makes PI3K inhibitors potential therapeutic options for EBVaGC patients that need further evaluation [52].

*ARID1A* is a component of the Switch/Sucrose Non-fermentable (SWI/SNF) chromatin remodeling complex and is a tumor suppressor gene [48,53]. *ARID1A* is frequently mutated in cancer and the majority of *ARID1A* mutations are inactivating mutations that cause a lack of ARID1A protein expression [48,54]. Most of the *ARID1A* mutations identified in EBVaGC are single nucleotide truncating mutations, resulting in the loss of ARID1A protein expression. However, some GC shows absent or weak protein expression despite the lack of detectable *ARID1A* mutations, suggesting that other epigenetic modifications, partially regulated by EBV-encoded micro RNAs (miRNAs), may contribute to ARID1A inactivation [55,56]. During DNA replication, ARID1A is involved in the recruitment of mismatch repair (MMR) protein MutS homolog 2 (MSH2) to chromatin, and its inactivation leads to an increase in gene mutations [53,54]. Loss of ARID1A expression is related to poor prognosis in GC patients [48,53,57,58].

*BCOR* encodes an anti-apoptotic protein, an epigenetic regulator involved in cell differentiation. *BCOR* mutations have been found in both solid and hematological tumors [59].

Amplification at 9p24.1 is frequently detected in EBVaGC [12]. This locus contains *JAK2*, *CD274* and *PDCD1LG2* leading to overexpression of JAK2, PD-L1 and PD-L2 respectively [12,48]. JAK2 is a receptor tyrosine kinase that regulates cell proliferation, differentiation, and apoptosis [50]. PD-L1 and PD-L2 are immunosuppressant proteins that act as negative regulators of T cell-mediated immunity. Moreover, EBV infection promotes PD-L1 expression through the activation of Interferon regulatory factor 3 (IRF3) via interferon-γ (IFN-γ) [60].

Besides a specific pattern of gene alterations, the comprehensive analysis of The Cancer Genome Atlas (TCGA) project showed that EBVaGC had a higher prevalence of DNA hypermethylation than any other type of cancer evaluated [12]. DNA methylation is involved in the regulation of gene expression and plays a crucial role in tumorigenesis [46]. Hypermethylation in the CpG DNA promoter of a gene suppresses its expression. Consistent with previous reports [61], the TGCA analysis confirmed that EBVaGCs show a specific methylation epigenotype, distinct from that found in GCs with high microsatellite instability (H-MSI). In particular, EBVaGC displayed hypermethylation of the *CDKN2A* (*p16^INK4A^*) promoter but not the *MutL protein homolog 1* (*MLH1*) promoter (typical of MSI-associated CIMP) [12]. Other genes silenced by DNA methylations in EBVaGC include genes involved in cell regulation, DNA repair, and apoptosis [48].

## 5. Clinical and Histopathological Features

There are no differences between the clinical presentation of EBVaGC and other types of GC. Early-stage disease is often asymptomatic, while common signs and symptoms in the advanced stage include dysphagia, asthenia, indigestion, vomiting, weight loss, early satiety, and iron deficiency anemia. Previous studies showed that EBVaGC has unique clinicopathological features with a predominance in men and younger individuals [19], though the male predominance decreases with age regarding risk estimates [20,62]. EBVaGCs frequently occur in the proximal part of the stomach (cardia and body) and generally show diffuse histological type [48]. Interestingly, several studies and meta-analyses reported a frequent EBV involvement in remnant gastric cancers, defined as tumors developing in the stomach after a previous partial gastrectomy for gastric ulcer or gastric carcinoma [15,19,63]. The higher prevalence of EBVaGC in remnant GCs, particularly in patients who underwent a Billroth II anastomosis, may be related to the chronic damage and the changes of the microenvironment made by pancreatic and bile juice reflux rather than to a more aggressive EBV variant in these subgroup of GCs [64,65].

An international pooled analysis including 4599 gastric cancer patients from 13 studies in Asia, Europe, and Latin America found that tumor EBV positivity was higher in early-stage GC [20]. By this data, a recent prospective observational study including 1146 metastatic GC Asian patients showed that the incidence rate of EBVaGC decreased with advanced TNM stage (9.3%,9.9%,6.7%, and 1.4% for Stage I, II, III, and IV, respectively) [66]. However, other large series did not confirm this higher prevalence in the lower stage [15,18]. The controversy concerning the relation between EBV positivity and lower cancer stage can be related to the fact that several Asian case series collected data on EBV positivity as part of screening programs thus explaining the higher incidence in the lower stage as compared to population-based data [67]. On the other hand, the excellent prognosis of EBVaGC in early or locally advanced stages [68,69] could explain the low incidence of this molecular sub-group in the metastatic setting. Macroscopically, on endoscopic observation, EBVaGC often forms ulcerated and depressed or saucer-like tumors with marked thickening of the gastric wall [48]. In the early stages, EBVaGC usually forms well-demarcated nodular lesions in the submucosa with less fibrosis than other gastric carcinomas [70]. Another characteristic feature is the multiplicity, which is the presence of multiple lesions occurring synchronously or metachronously in the stomach [71,72,73].

**Figure 1:**
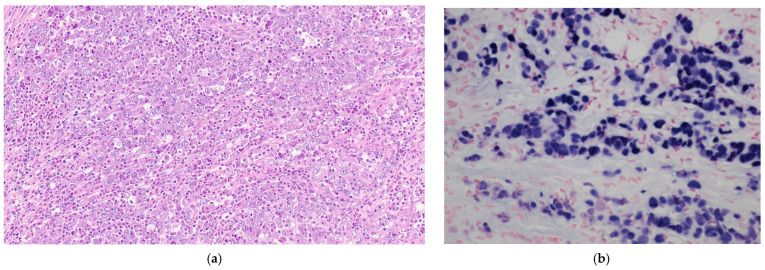
(**a**) EBV-associated gastric carcinoma exhibiting a typical lymphoepithelioma-like carcinoma morphology; (**b**) Tumor cells of an EBV-associated lymphoepithelioma-like carcinoma showing strong EBER reactivity (EBER in-situ hybridization).

Histologically, there are three types of EBVaGC: LELC-type (or medullary type), conventional-type adenocarcinoma, and carcinoma with Crohn’s disease-like lymphoid reaction (CLR) [74]. LELC-type is a poorly differentiated carcinoma characterized by small clusters of tumor cells with unclear tumor-stroma boundaries and with dense infiltration of lymphocytes (Figure 1a) [75].

The conventional-type adenocarcinoma is well-moderately differentiated with a variable amount of infiltrating lymphocytes and morphologically is similar to EBV-negative gastric carcinoma. The CRC sub-type has a morphology intermediate between the LELC-type and conventional-type adenocarcinoma. Histologically, this type is characterized by three or more lymphoid follicles with active germinal centers at the advancing edge of the tumor and frequent tubule or gland formation. There are fewer lymphocytes compared to tumor cells and minimal or no desmoplasia [74].

Regardless of different morphological subtypes, EBVaGC is often morphologically identical to EBV-negative GC; therefore, identifying the presence of EBV in carcinoma cells is essential to define EBVaGC cases. The gold standard for identifying EBV infection is in situ hybridization (ISH) to detect EBV-encoded RNA (EBER), which is abundant in infected cells (Figure 1b) [11]. The EBER-1 probe used in ISH can be applied to formalin-fixed and paraffin-embedded gastric cancer specimens, enabling the detection of EBV with accurate localization and strong specificity [76]. Genome sequencing or ddPCR are potential alternative techniques to identify EBV-positive gastric tumors. Still, they are expensive and time-consuming, thus limiting their applicability as a primary screening method for EBV in cases of conventional-type adenocarcinomas [77].

On histological examination, most cases of GC are positive for CDX2, though the expression of CDX2 and MUC2 is significantly lower in EBVaGC compared to EBV-negative GCs [78]. Another attractive characteristic is that >80% of EBVaGC cases express Claudin 18 (CLDN18), while Claudin 3 (CLDN3) expression is infrequent (5%) [79]. The expression of CLDN18 is specific for the normal stomach and lung, while CLDN3 is generally expressed in the normal intestine but not in the normal stomach. Hence, EBVaGC demonstrates traits identical to those of mature or immature gastric epithelium but somehow different from classic GC, and this characteristic might be challenging when the histological diagnosis has to be made on metastatic lesions [80].

It is also worth noting that about 50% of EBVaGC are PD-L1 positive in tumor tissue by immunohistochemistry [81]. The higher PD-L1 overexpression in this type of GC is due to high levels of CD274 focal amplification [12,48]. Positive PD-L1 expression is associated with less aggressive clinical and pathological features, predicted superior prognosis, and better efficacy of immunotherapy in EBVaGC [48].

## 6. Treatment Options and Future Direction

### 6.1. Chemotherapy

There has been no systematic investigation about the responsiveness of advanced EBVaGCs to chemotherapies, and no particular regimens have been tested for treating this subgroup of GC in prospective clinical trials. Some in vitro studies reported that EBV-positive GC cell lines have higher chemoresistance to docetaxel and 5-fluorouracil than EBV-negative ones [82,83]. However, retrospective series reported disease control rates ranging from 90.3% to 100% among EBVaGC patients treated with fluoropyrimidine and platin-based first-line therapy and significantly better survival than EBV-negative patients [22,68]. All available evidence is limited by the retrospective nature and the small sample size, so prospective studies with larger samples are warranted to confirm these findings.

### 6.2. Immunotherapy

The unique clinical and molecular characteristics of EBVaGC indicate a close interaction between the cancer and the immune system. EBVaGCs often show a dense infiltrate of lymphocytes, seen in both undifferentiated (LELC) and typical gastric adenocarcinoma cases. This trait is associated with EBV’s ability, compared to other human viruses, to hyper-activate the cellular immune response, as shown by the elevated CD8+ T cell response observed in patients with infectious mononucleosis [24]. Besides the EBV infection’s direct immunogenicity, EBVaGCs frequently harbor copy-number amplifications of PD-L1 and PD-L2- that induce immune tolerance by activating the PD-1 pathway to inhibit immune checkpoints [12,84]. Moreover, recent evidence suggests that some of the mature microRNAs encoded by EBV, directly and indirectly, upregulate the expression of PD-L1, promoting tumor immune escape [85]. In line with these findings, gene expression data from the TCGA GC cohort revealed that EBV-positive tumors have higher immune checkpoint pathway (PD-1, CTLA-4 pathway) expression than any other type of cancer and dysregulation of immune cell signaling molecules [12,86].

Although a strong rationale suggests the efficacy of immune checkpoint inhibitors (ICIs) in the EBV-positive molecular subgroup, specific evidence from immunotherapy trials in this population is limited, and their efficacy was equivocal.

Kim et al. reported an overall response rate (ORR) of 100% in 6 EBVaGC patients treated with pembrolizumab as salvage treatment in a phase II clinical trial [87]. However, in a phase 2 trial evaluating the safety and efficacy of camrelizumab (an anti-PD-1 antibody) as a salvage treatment in 6 EBVaGC patients, no patient achieved an objective response even if a disease control rate of 67% was reported [88]. Other recent observational studies or subgroup analyses of clinical trials showed ORRs ranging from 0 to 100% in EBVaGC treated with ICIs [86,89,90,91,92,93,94,95,96] (Table 1). Notably, all six patients responsive to ICIs reported by Kim et al. had positive PD-L1 expression in tumor tissues. To investigate the potential impact of PD-L1 expression on the efficacy of ICIs in EBVaGC patients, a recent review analyzed data from 39 patients from 8 reports (3 prospective studies, four retrospective studies, and one case report). The analysis showed that PD-L1 positive patients had a better median progression-free survival (mPFS) compared to PD-L1 negative ones [97]. Moreover, the analysis of public genomic mutation and transcriptome profile datasets of EBVaGC showed enhanced immune-related signal pathways in PD-L1 high EBVaGC. These data suggest that PD-L1-positive EBVaGC could be considered as a specific subgroup with a ‘hot’ immune microenvironment and higher sensitivity to anti-PD-1 immunotherapy. However, this hypothesis must be assessed in a prospective clinical setting to draw definitive conclusions.

An alternative immunotherapeutic approach explored in EBVaGC involves the use of adoptive immunotherapy, based on the adoptive transfer of gene-engineered T cells to induce tumor regression [98,99]. The rationale for applying adoptive immunotherapy for EBVaGCs is that all EBV-associated tumors involve viral latency, and some of the products of viral latent genes are highly immunogenic [98]. Studies exploring the efficacy of the infusion of virus-specific T cells in patients with EBV-associated lymphoma and NPL showed impressive clinical responses [100,101,102]. Unfortunately, EBVaGCs arising in immunocompetent persons express a more limited array of EBV-specific antigens, showing a lower immunogenic activity. However, studies exploring this therapeutic strategy in EBVaGCs and other EBV+ malignancies are still ongoing (Table 2). They may provide a new option for treating EBV-positive recurrent cancer patients resistant to conventional therapies.

### 6.3. Target Therapies

As previously discussed, the comprehensive analysis conducted by the TCGA showed that EBVaGC is characterized by a unique genetic pattern [12]. Some of the more frequently reported gene alterations or one of their downstream pathways might be potential therapeutic targets for EBVaGCs. Interestingly, about 80% of EBVaGC patients display activating mutations in PIK3CA, suggesting a crucial PI3K/AKT signaling role in this molecular subgroup. PI3K or dual PI3K/mTOR inhibition has been tested preclinically in gastric cancers and showed promising therapeutic results in EBVaGC cell models [103,104,105,106]. Several PI3Ka-mutant selective inhibitors have been tested in clinical trials. However, the activity and efficacy in the specific subgroup of EBVaGCs need further investigation (NCT04526470).

ARID1A is the second most frequently mutated gene in EBVaGCs. Because mutations or the epigenetic inactivation of ARID1A may drive cancer development in EBVaGC, designing target therapies against ARID1A in EBVaGC would be fascinating. Unfortunately, as a tumor suppressor gene, ARID1A is a poor therapeutic target. However, the loss of function of ARID1A has been shown to interact with numerous signaling pathways involved in the oncogenesis: the DNA damage repair machinery, the PI3K/AKT/mammalian target of rapamycin (mTor) pathway, the KRAS pathway, and enhancer of zeste homolog 2 (EZH2) pathway [107]. Targeting these pathways may be effective in EBVaGC. In particular, one of the most intriguing strategies recently proposed is testing specific inhibitors against the histone methyltransferase EZH2.

EZH2 is a methyltransferase that tri-methylates histone H3, silencing gene expression [108]. Previous studies reported that ARID1A and EZH2 bound the promoter of the gene encoding phosphoinositide-3-kinase interacting protein 1 (PIK3IP1), which negatively regulates PI3K-Akt signaling [109]. ARID1A activates PIK3IP1 expression and usually dominates over EZH2, which suppresses PIK3IP1. When ARID1A is absent, EZH2 silences PIK3IP1 thus activating the PI3K-Akt pathway. Interestingly, preclinical models showed that EZH2 inhibitor administration decreases the viability of ARID1A-deficient gastric cells in a dose-dependent manner, therefore suggesting that suppression of EZH2 activity may serve as a synthetic lethal therapeutic strategy to target ARID1A-mutated cancers [110]. Apart from EZH2 inhibition, other treatment strategies based on synthetic lethality, such as poly ADP-ribose polymerase (PARP) inhibitors, could be effective in EBVaGCs. ARID1A facilitates the efficient processing of double-strand breaks to single-strand ends; as a consequence, ARID1A alterations interfere with DNA damage repair. In vitro and in vivo models show that ARID1A deficiency sensitizes cancer cells to PARP inhibitors [111,112]. However, the efficacy of PARP inhibitors in patients with ARID1A alterations is not yet clear, and further research in gastric cancer models is necessary.

## 7. Prognosis

Many studies have attempted to assess whether patients with EBVaGC have a better prognosis than those with EBV-negative subtypes, drawing different conclusions. Applying a robust, highly sensitive, and specific prediction model to categorize according to the 4 TGCA subtypes a cohort of 267 GC from the MD Anderson Cancer Center, Sohn et al. reported that the EBV subtype was associated with the best prognosis for both relapse-free survival and overall compared to the other TGCA subtypes [113]. This observation aligns with similar data obtained in GC patients with resectable early-stage disease [114,115]. In contrast, Li et al. recently reported no statistically significant difference in survival between EBVaGC and EBV-negative cases in a retrospective cohort of 1031 consecutive GC patients [116]. However, among EBVaGC patients, those with the LELC subtype had the best prognosis compared to Crohn’s-like lymphocytic reaction (CLR) and conventional adenocarcinoma (CA) subtypes. An international pooled analysis including 4599 GC patients from 13 studies showed that, even when adjusted for stage and other prognostic confounders, EBV positivity was associated with lower mortality [20]. However, further evidence is needed to conclusively determine the effect of EBV infection on EBVaGC patient survival.

## 8. Discussion

After the report by Kim et al. of the exceptional responsiveness of some EBVaGC cases to anti-PD-L1-based immunotherapy [87], extensive research focused on understanding the factors influencing the response of EBVaGC to immunotherapy. Efforts have also been made to improve our understanding of the epidemiology and pathogenesis of EBVaGC through new studies and meta-analyses. In light of this, our paper provides an overview of the most recent knowledge on the epidemiology and clinic-pathologic features of EBVaGC. It also discusses the various hypotheses regarding the carcinogenic mechanisms resulting from the intricate interaction between EBV and gastric mucosa. Furthermore, the review elaborates on the relevant treatment strategies that have recently been evaluated or are currently being explored for this subtype of gastric cancer. The goal is to offer a comprehensive understanding of the disease and its potential future directions. Unfortunately, the existing data is insufficient to comprehend the mechanisms underlying the development of EBVaGC fully and to draw definite conclusions about the prognostic and predictive impact of EBV positivity in perioperative and advanced-stage scenarios. Standard treatment strategies for patients with EBVaGC have yet to be established and further research is needed.

Compared to previous reviews on this field, our review stands out for presenting the latest epidemiological data on EBVaGC and a summary of existing and new theories on the interaction mechanisms between EBV and gastric mucosa that lead to the development of gastric cancer. Our paper is also the first, to our knowledge, to elaborate separately on the prevalence of the two most important pathological subtypes of EBVaGC, namely, the “lymphoepithelioma-like” type and the “conventional” type. Additionally, we provide a comprehensive review of the available evidence on treatment options for EBVaGC and ongoing trials in this area.

Our work has some limitations: first, the lack of universal screening for EBV among GC patients means that most of the epidemiologic and prognostic data in our review come from retrospective series, which may limit their reliability. Second, the paper included many studies with small sample sizes, which makes it difficult to draw definitive conclusions about the effectiveness of different treatment strategies for EBVaGC. However, it should be acknowledged that the relatively low prevalence of this subgroup makes it challenging to design large prospective randomized studies dedicated to this topic. Third, our review may not have included some future treatment strategies, and ongoing trials addressing EBVaGC may not be cited, as they do not explicitly include EBV evaluation in their inclusion criteria or pre-planned subgroup analyses.

In summary, implementing universal screening for EBV among GC patients could provide greater insight into the clinical and prognostic features of EBVaGC. Additionally, ongoing studies are leveraging the unique molecular characteristics of EBVaGCs to investigate different treatment strategies, with the potential to offer effective treatment options in the future. Although there is still much to learn about the features and treatment options for EBVaGC, available data indicates that gaining a better understanding of this distinctive subgroup of GC could potentially revolutionize treatment approaches for a significant portion of GC patients.

## Figures and Tables

**Table 1:** Published studies evaluating immune checkpoint inhibitors against EBVaGC.

Reference	Phase	Target Population	Number of EBVaGC pts	Immunotherapy Regimen	ORR (%)	PFS (Months)	OS (Months)
Kim et al., 2018 [87]	II	Stage IV GOJ or GC pts treated with at least 1 line of chemotherapy and naive to anti-PD-1, anti-PD-L1, or anti-PD-L2 antibodies.	6	Pembrolizumab	100.0%	8.5	NR
Panda et al., 2018 [86]	I	Metastatic or locally advanced solid tumors, who failed or were not candidable to standard therapies	1	Avelumab	100.0%	NR	NR
Mishima et al., 2019 [88]	Observational	Advanced GC pts who were treated with nivolumab after two or more chemotherapy regimens	4	Nivolumab	25.0%	NR	NR
Wang et al., 2019 [89]	Ib/II	Patients with advanced GC, esophageal SCC, NPC, and head and neck SCC	4	Toripalimab	25.0%	NR	NR
Kim et al., 2020 [90]	Observational	Advanced GC patients treated with pembrolizumab or nivolumab	4	Nivolumab/ Pembrolizumab	50.0%	NR	NR
Kubota et al., 2020 [91]	Observational	Locally advanced, or metastatic GC patients treated with an anti PD-1 inhibitor after standard chemotherapies	6	PD-1 inhibitors	33.3%	3.7	NR
Kwon et al., 2020 [92]	Observational	Stage IV GC patients treated with PD-1 inhibitors	7	Nivolumab or Pembrolizumab	42.9%	NR	NR
Xie et al., 2020 [93]	Observational	Stage IV EBVaGC patients treated with ICIs as the first-, second-, or third-line therapy	9	PD-1 inhibitors, PD-1 inhibitors +XELOX, nivolumab + ipilimumab	33.3%	NR	NR
Sun et al., 2021 [94]	II	Metastatic or unresectable HER2-, pMMR, EBVaGC pts not previously treated with ICIs who failed or were intolerant to or prior chemotherapy	6	Camrelizumab	0%	2.2	6.8
Bai et al., 2022 [95]	Observational	Locally advanced or metastatic pMMR EBVaGC patients treated with an anti PD-1 inhibitors	22	PD-1/PD-L1 inhibiros (8 pts) or combination of anti-CTLA-4 plus anti-PD-1/L1 (14 pts)	54.5%	NR	NR
Duan et al., 2024 [96]	Observational	Metastatic GC pts who underwent various PD-1 inhibitor-based treatments as first-line therapy	6	PD-1 inhibitors +/−chemotherapy	NR	17.0	28.0

EBVaGC: Epstein-Barr virus-associated gastric cancer; GC: gastric cancer; GOJ: gastroesophageal junction; HER2-: human epidermal growth factor receptor 2 negative; ICI: immune checkpoint inhibitor; NPC: nasopharyngeal carcinoma; NR: not reported; ORR: overall response rate; OS: overall survival; PD-1: programmed cell death protein 1; PD-L1: programmed cell death protein ligand 1; PD-L2: programmed cell death protein ligand 2; PFS: progression-free survival; pMMR: mismatch repair proficient; pts: patients; SCC: squamous cell carcinoma.

**Table 2:** Ongoing studies evaluating different therapeutic options against EBVaGC.

Study Title (Clinical Trial Registration Number)	Phase	Target Population	Setting of Disease	Treatment Regimen	Primary End-Point	Recruitment State
Immunotherapy in MSI/dMMR Tumors in Perioperative Setting (IMHOTEP) (NCT04795661)	II	Non-metastatic, resectable-EBV+ GC, MSI/dMMR CRC, esophago-gastric cancer, endometrial carcinoma, biliary tract adc, pancreatic adc, and small bowel adc.	Localized	Pembrolizumab *	pCR	Ongoing
D-1 Knockout EBV-CTLs for Advanced Stage Epstein-Barr Virus (EBV) Associated Malignancies (NCT03044743)	I/II	Stage IV EBV+ NPC, lymphoma or stage IV GC	Advanced stage	PD-1 knockout EBV-CTLs	Safety	Unknown
Nanatinostat Plus Valganciclovir in Patients With Advanced EBV+ Solid Tumors, and in Combination With Pembrolizumab in EBV+ RM-NPC (NCT05166577)	Ib/II	Recurrent or metastatic EBV+ NPC (experimental choort) and advanced/metastatic EBV+ non-NPC solid tumors (exploratory proof-of-concept cohort only)	Advanced stage	Nanatinostat **+valganciclovir vs. Nanatinostat + valganciclovir + pembrolizumab **	Safety (Ib) ORR (II)	Ongoing
A Study to Evaluate Serplulimab in Combination with Docetaxel +S-1 VS. Docetaxel +S-1 as Adjuvant Treatment Therapy in Stage IIIc Gastric Cancer (NCT05769725)	II	PD-L1+ or MSI-H or dMMR or EBV+ GC patients	Stage IIIC	Docetaxel + S1 + Serplulimab (HLX10) *	DFS	Ongoing
Neoadjuvant Immunotherapy and Chemotherapy for Locally Advanced Esophagogastric Junction and Gastric Cancer Trial (NICE) (NCT04744649)	II	GC patients with one of the following: - PD-L1 CPS ≥ 5. (experimental group) - EBV+ (exploratory group). - dMMR (exploratory group) - MSI-H (exploratory group)	cT3/4a Nx or T2 N+, M0 (AJCC 8th)	XELOX or SOX vs. XELOX or SOX + JS001 (toripalimab) *	MPR (residual tumor defined as less than 10%)	Ongoing
A Phase II Study of Preoperative Pembrolizumab for Mismatch-Repair Deficient and Epstein-Barr Virus Positive Gastric Cancer Followed by Chemotherapy and Chemoradiation With Pembrolizumab (NCT03257163)	II	Resectable dMMR or EBV+ GC patients	Stage Ib-IIIC	Pembrolizumab (2 cycles) followed by surgery, followed by pembrolizumab +capecitabine (5 cycles), followed by pembrolizumab (11 cycles) and radiotherapy (5 weeks).	RFS rate	Ongoing
A Multi-center, Single-arm, Open, Phase I/IIa Clinical Trial to Evaluate the Efficacy and Safety of EBViNT Cell (EBV Specific Autologous CD8+ T Cell) in Patients With Treatment Failed Epstein Barr Virus (EBV)-Positive Malignancies (NCT03789617)	I/IIa	EBV+ Extranodal NK/T-cell lymphoma, and EBV+ GC or esophageal adenocarcinoma	Advanced stage	EBV specific autologous blood-derived T lymphocytes	Safety ORR	Ongoing
A Study of a Selective T Cell Receptor (TCR) Targeting, Bifunctional Antibody-fusion Molecule STAR0602 in Participants With Advanced Solid Tumors (START-001) (NCT05592626)	I/II	TMB-H tumors, or MSI/dMMR tumors, or virally associated tumors, or metastatic triple negative breast cancer, or relapsed and refractory epithelial ovarian cancer, or metastatic castration-resistance prostate cancer, or KRAS wild type CRC, or KRAS mutant CRC, or stage IV NSCLC	Advanced stage	STAR0602 ***	Safety and ORR	Ongoing

* anti-PD-1 antibody; ** histone deacetylase inhibitor; *** Bifunctional antibody-fusion molecule that selectively activates and expands a sub-set of human aβ T cells expressing the germline-encoded variable b6 and b10 regions of the T cell receptor; Adc: adenocarcinoma; CRC: colorectal cancer; CPS: combined positive score; CTLs: cytotoxic T lymphocytes; DFS: disease-free survival; dMMR: deficient mismatch repair; EBV+: EBV-positive; GC: gastric cancer; MSI microsatellite instable; MPR: Major pathologic response; NPC: nasopharyngeal carcinoma; NSCLC: non-small cell lung cancer; pCR: pathologic complete response; RFS: Recurrence-free survival; TMB-H: tumor mutational burden high.

## Data Availability

Not applicable.
